# Consistency and individuality of honeybee stinging behaviour across time and social contexts

**DOI:** 10.1098/rsos.241295

**Published:** 2025-01-29

**Authors:** Kavitha Kannan, C. Giovanni Galizia, Morgane Nouvian

**Affiliations:** ^1^ Department of Biology, University of Konstanz, Konstanz, Germany; ^2^ Center for the Advanced Study of Collective Behaviour, University of Konstanz, Konstanz, Germany; ^3^ International Max Plank Research School for Quantitative Behaviour Ecology and Evolution, Konstanz, Germany; ^4^ Zukunftskolleg, University of Konstanz, Konstanz, Germany

**Keywords:** consistency, inter-individual variability, defensive behaviour, honeybees, alarm pheromone, social modulation

## Abstract

Whether individuals exhibit consistent behavioural variation is a central question in the field of animal behaviour. This question is particularly interesting in the case of social animals, as their behaviour may be strongly modulated by the collective. In this study, we ask whether honeybees exhibit individual differences in stinging behaviour. We demonstrate that bees are relatively stable in their decision to sting—or not—in a specific context and show temporal consistency suggestive of an internal state modulation. We also investigated how social factors such as the alarm pheromone or another bee modulated this behaviour. The presence of alarm pheromone increased the likelihood of a bee to sting but this response decayed over trials, while the presence of a conspecific decreased individual stinging likelihood. These factors, however, did not alter stinging consistency. We therefore propose that social modulation acts by shifting the stinging threshold of individuals. Finally, experimental manipulation of group composition with respect to the ratio of aggressive and gentle bees within a group did not affect the behaviour of focal bees. Overall, our results establish honeybee stinging behaviour as a promising model for studying mechanistically how collective and individual traits interact to regulate individual variability.

## 1. Introduction

Social collectives, such as honeybee colonies, are formed by many individuals with different tasks. Individuals vary in their propensity to display a particular behaviour [1], say collect pollen, nectar or water, fan air to cool the hive in summer, or attack an intruder and sting. The response-threshold model postulates that diversity in response thresholds creates diversity across individuals, which in turn allows for a robust and stable group response [2]. In bees, many behaviours are regulated by age polyethism: young bees are nurses and older bees are foragers. For some other behaviours, however, age is but one criterion. In particular, guarding and stinging propensities seem to vary between individuals of the same age group [3,4]. It is unknown, however, whether this form of task allocation is a short-term, ‘ad hoc’ organization possibly controlled by collective signals such as pheromones (i.e. any bee could be a docile or an aggressive bee in different situations), or rather an individual trait whereby some bees are consistently more or less aggressive than others.

Colonies can vary enormously in their defensive behaviour, and selective breeding demonstrated early on the heritability of this trait [5,6]. Several studies further identified genomic regions associated with colony-level defensive behaviour [7–9]. However, linking individual and colony phenotypes has proven challenging, with only a single locus being successfully associated with stinging behaviour at both levels [8,10,11]. Both cross-fostering experiments and a genomic study confirmed that the social environment strongly influences the expression of individual aggressiveness in a normative way [12–14]. The mechanisms at play seem to involve a broad modulation of brain gene regulatory networks rather than variations in gene expression [15].

On top of this long-term regulation, other social factors influence whether individual bees take the decision to sting or not during a particular defensive event. First and foremost, direct communication occurs through release of the sting alarm pheromone (SAP) by stinging bees [16]. The SAP is a complex mixture of over 40 compounds but its main component, iso-amyl acetate (IAA), is sufficient to elicit most of the behavioural response [17]. The concentration of this pheromone indicates how many bees are already involved in the attack: as more and more bees detect and sting the predator, the SAP concentration increases. This can be interpreted as a confirmation of the presence of a danger by many nestmates, giving more credibility to this social signal. Indeed, in this regime, the likelihood of a bee to sting increases with SAP concentration. After a certain point, however, the correlation between SAP concentration and stinging likelihood becomes negative. This may be a strategy that prevents over-stinging [18]. The second social factor that seems to regulate individual aggressiveness is group size: in larger groups, honeybees are less likely both to initiate an attack and to respond (by stinging) to a given SAP concentration [19]. Finally, there is some evidence that group composition might affect the defensive behaviour of individual honeybees [20,21] and termites [22].

The complex interactions between social factors at different timescales and their impact on the expression of behavioural traits are particularly interesting, since both the individual phenotype and the collective level need to be considered [23–25]. But how to look in individuals for a phenotype that seems to only appear at the collective level? Comparisons between workers issued from different colonies have limited power because of the number of confounding factors that they inherently include. For example, colony defensiveness has been linked to several other collective traits such as foraging activity or the tendency to repair combs [26]. Within workers of the same colony, differences in aggressiveness may be hard to disentangle from other variables such as age and task [4,14,27,28]. It is an open question, therefore, whether individuals themselves fall into ‘aggressive’ and ‘gentle’ categories, or whether their aggressiveness is entirely controlled by the collective.

In this study, we set out to evaluate whether honeybees exhibit stable inter-individual variability in defensive behaviour, by repeatedly testing the same individuals in a stinging assay. Furthermore, we investigated how social factors such as the presence of another individual and/or of the SAP affects the expression and stability of stinging behaviour. Finally, we evaluated how bees adjust their stinging behaviour depending on the aggressive tendencies of their social partners. We show that individual bees have a stable baseline stinging behaviour, which is further modulated in a situation-dependent manner by the social environment.

## 2. Methods

We designed two different experiments using an established stinging assay [29]. The ‘Consistency’ experiment focused on the question of inter-individual variability and its modulation by social factors. The ‘Group composition’ experiment tested whether the phenotype of other group members influences the stinging behaviour of focal individuals.

### 2.1. Experiment 1: consistency

#### 2.1.1. Honeybees

Bees were collected from 10 honeybee colonies (*Apis mellifera carnica*, *Apis mellifera mellifera*, and hybrids of both sub-species; electronic supplementary material, table S1) housed at the University of Konstanz (Konstanz, Germany) in their active season (May–October) of the years 2022 and 2023. Defensive bees were collected from the hive by waving a large black ostrich feather in front of the colony for a few seconds, as in previous studies (e.g. [19,29]). Bees were then placed into a *Ziploc* bag and immediately chilled on ice for about 5 minutes, before marking and placing them in modified 50 ml syringe tubes. They were then fed with 50% (v/v) sugar water ad libitum and allowed to recover for 15 minutes.

#### 2.1.2. Stinging assay

After the recovery time, bees were tested either individually or in pairs for their stinging responsiveness in a stinging assay [29]. Briefly, the bees were introduced into a closed cylindrical arena made of clear plastic (diameter 14 cm and height 4 cm) and exposed to a rotating three-dimensional printed dummy with a feather attached to one end. Unlike in previous studies, we did not cover the dummy with a leather piece so that bees would not lose their stingers upon attacking. Odours were delivered from three entry points on the side of the arena by placing a filter paper soaked with 10 μl of odorant within a falcon tube through which an air flow (60 ml min^−1^) was carried. The solvent was mineral oil (MO; Acros Organics, CAS: 8042-47-5) which acted as a control while the odour (IAA; Sigma-Aldrich, CAS: 123-92-2, 10% (v/v) in MO) is the main active compound in the honeybee SAP [17]. The arena lid had holes to avoid the building up of odour inside the arena, and an air exhaust was placed next to the arena to clear the area of lingering odours. Stinging behaviour was identified visually from the characteristic U-shaped posture of bees, where the bee bit with its mandibles while pressing the tip of its abdomen on the dummy, in an apparent attempt to sting. Video recordings of each experiment were collected for off-line verification purposes. Each trial lasted 3 minutes, at the end of which each bee received a score of either 0 (no stinging) or 1 (at least one stinging attempt). We also recorded the reaction time as the delay between the start of the experiment (bees entering the arena) and the attack, and the stinging order in pairs of bees. After each trial, the arena was wiped clean with 70% ethanol.

#### 2.1.3. Protocol

**Figure 1.**
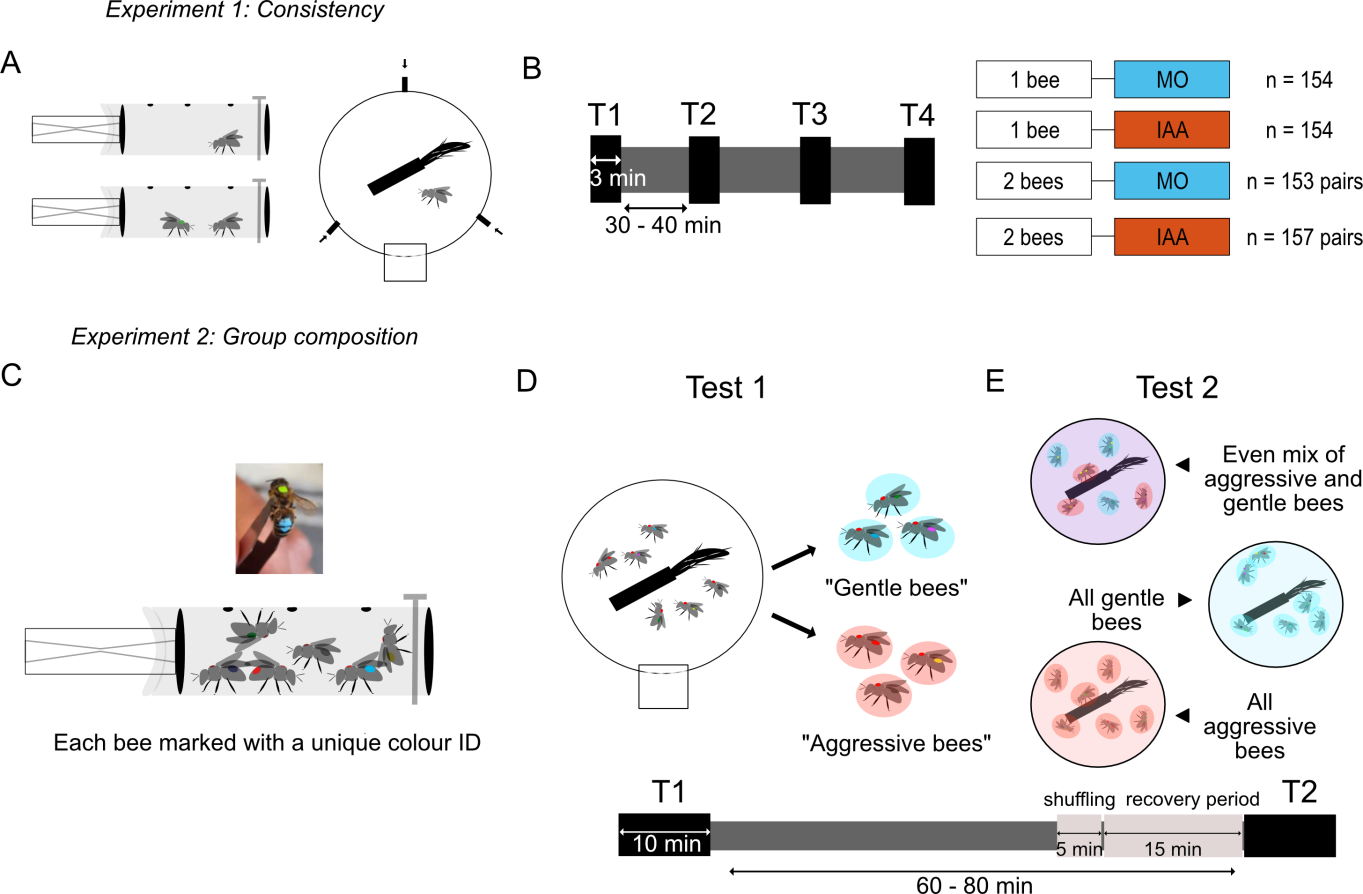
Experimental methods. (*a*,*b*) Consistency experiment. (*a*) One or two bees were placed in modified syringes (left), then tested in the stinging assay with a rotating dummy (right). Arrows indicate odour entry points for iso-amyl acetate (IAA), a sting alarm pheromone compound, or the solvent control mineral oil (MO). (*b*) Bees were tested repeatedly on four consecutive trials (T1–T4), each lasting 3 minutes, with an intertrial interval of 30–40 minutes (left). We tested four conditions (2 group sizes × 2 odours; right). (*c*–*e*) Group composition experiment. (*c*) Individual bees were marked with a unique colour ID on their thorax and abdomen and placed in syringes in groups of six. (*d*) Each group of bees was tested a first time (test duration: 10 minutes), and individuals were classified as ‘aggressive’ or ‘gentle’ if they stung or not, respectively. (*e*) Following the first test of all available groups, bees were cooled down and shuffled on ice, to create three types of group: even mix of aggressive and gentle, all-gentle and all-aggressive groups. After a recovery period of 15 minutes, bees were tested a second time in these new groups.

We tested two group sizes: either a single bee or a pair of bees (in which case one bee was marked with water-based paint (Posca markers) for identification). An experimental session always included four pairs of bees and four individual bees from a single colony. Half were tested with MO, the other half with IAA, thus resulting in four conditions (tested twice per session): (i) single bees with solvent control (1-MO), (ii) single bees with alarm pheromone (1-IAA), (iii) pairs of bees with solvent control (2-MO) and (iv) pairs of bees with alarm pheromone (2-IAA; figure 1*a*). The data from pairs of bees were analysed either as a collective output (labelled pair-MO and pair-IAA) or as the output of each individual within the pairs (labelled ind-MO and ind-IAA). These groups were tested four times (trials T1, T2, T3 and T4), with an inter-trial interval of 30 to 40 minutes (figure 1*b*). Importantly, each bee/pair was exposed to the same odour in all four trials. At the end of each trial, the bees were collected in the same tube and fed with sugar water ad libitum. Our sample size was at least 15 per colony and per condition, thus totalling around 150 single bees or pairs of bees for each condition (see electronic supplementary material, table S1, for a detailed overview). If a bee died or exhibited signs of impaired locomotion (slow or clumsy walk, inability to hold upside-down or to roll back on its feet after falling), all its trials were excluded from analysis. If this bee belonged to a pair, the pair as a whole was excluded.

### 2.2. Experiment 2: group composition

#### 2.2.1. Honeybees

For this experiment, defensive bees were collected from seven honeybee colonies as in the other experiment, during the summer months of 2021−23 (electronic supplementary material, table S2). After cold-anaesthesia treatment for 5 minutes, bees were transferred to a glass container placed on top of an ice box. Bees were individually marked with a unique colour code on their thorax and abdomen and placed in groups of six in the modified syringe tubes (figure 1*c*). The bees were fed with sugar water ad libitum and allowed to recover for 15 minutes.

#### 2.2.2. Stinging assay and changing group composition

The stinging assay was the same as the one used for the ‘Consistency’ experiment and described above, except that the test duration was increased to 10 minutes to ensure that all bees had enough time to react even in the case of successive recruitment waves. No odours were introduced in the arena for this experiment. After a first test in groups of 6, each bee was classified as aggressive or gentle depending on its behaviour (i.e. whether it stung or not; figure 1*d*). Stinging behaviour was recognized and scored as before. In addition, we recorded the stinging order within a group (with rank 0 for the first bee(s) to sting) and the reaction time of each bee in the group. Note that if multiple bees attacked simultaneously, they were given the same rank and the following bee(s) received the rank corresponding to the number of bees already stinging (e.g. the sequence could be [0, 0, 2, 2, 4, 5]). This way, the rank of each bee corresponds to the number of alarm pheromone units released in the arena at the time that it stung. At the end of the trial, bees from the arena were collected back in the same syringe and were fed with sugar water ad libitum until the end of all first tests.

Following this, the syringes were placed in a box of ice for 5 minutes to cool down the bees so that they could be redistributed into new groups. We grouped bees into one of three group types: a mixed group (3 aggressive bees and 3 gentle bees); an all-aggressive group (6 aggressive bees); or an all-gentle group (6 gentle bees). The bees were once again fed with sugar water and given 15 minutes to recover before the start of test 2. Identical behavioural observation and scoring was performed in test 2 as in test 1.

### 2.3. Statistics

#### 2.3.1. Consistency experiment

**Figure 2.**
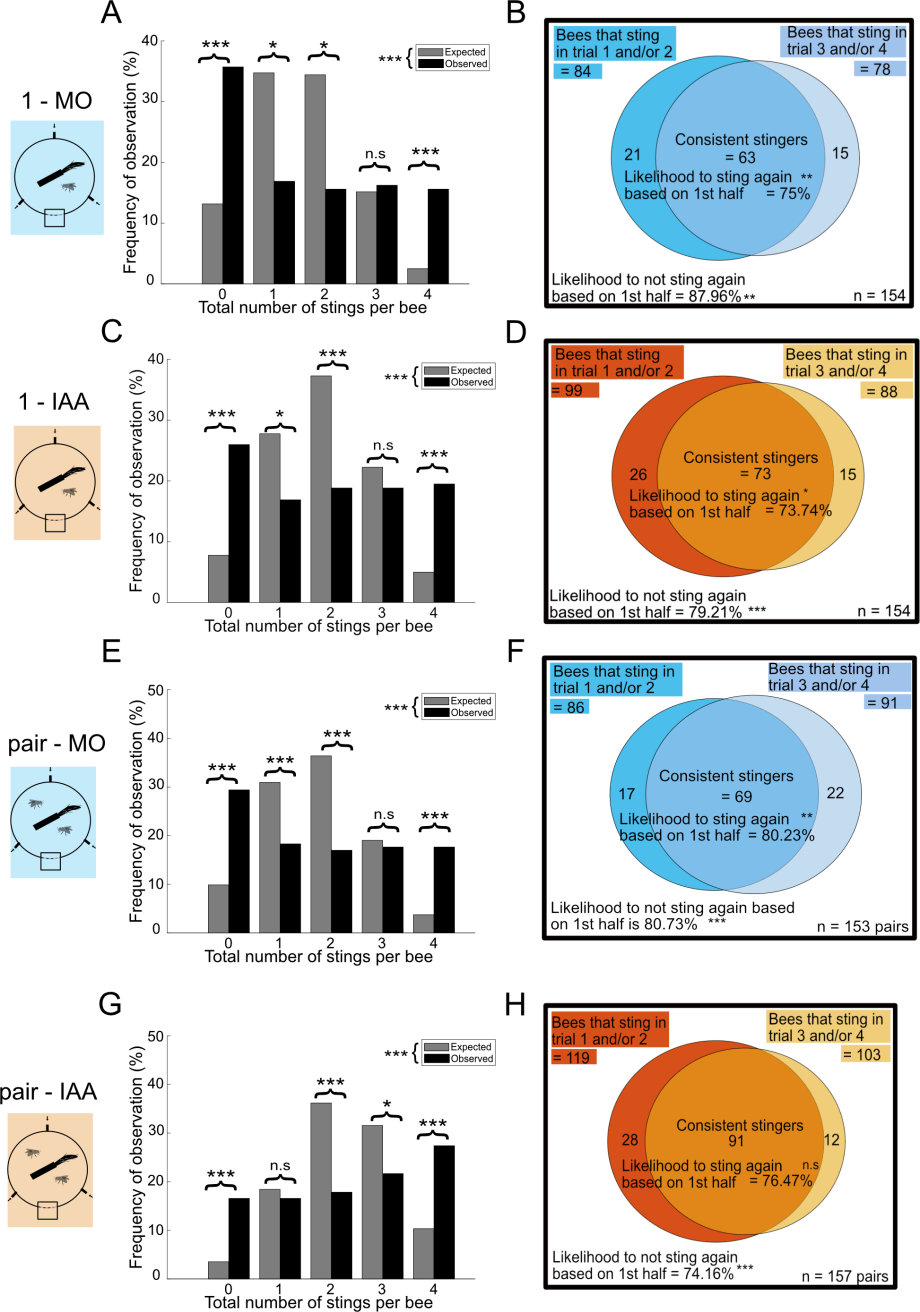
Inter-individual variability and consistency in stinging behaviour. (*a*,*b*) Single bee—MO condition; (*c*,*d*) single bee—IAA condition; (*e*,*f*) paired bees—MO condition; (*g*,*h*) paired bees—IAA condition. (*a*,*c*,*e*,*g*) Observed (black) and expected (grey) distributions of stings per bee. The expected distribution was calculated using binomial probability rules given the average stinging frequency observed in the dataset considered. The observed distribution did not fit the expected distribution in any conditions, indicating significant inter-individual differences in stinging likelihood. Pairwise comparisons for each number of stings are also indicated. (*b*,*d*,*f*,*h*) Venn plots showing the number of bees stinging in the first half of the experiment (trials 1 and/or 2), in the second half of the experiment (trials 3 and/or 4) and their overlap. The likelihood to sting (or not) again was compared to the assumption that both halves of the experiment were independent. Significant differences indicate behavioural consistency. All statistical comparisons were done with *χ*^2^ tests; n.s. *p* > 0.05, *p < 0.05, **p < 0.01, ***p < 0.001.

Most of our analysis was done in MATLAB v9.9.0 (R2020b). To look for inter-individual variability in stinging frequency, we compared the observed distribution to a theoretical distribution in which all bees would have the same probability to sting (figure 2*a,c,e,g*; electronic supplementary material, figure S1). We calculated this overall stinging probability *p* in each of the six datasets (1-MO, 1-IAA, pair-MO, pair-IAA, ind-MO and ind-IAA) by dividing the total number of stinging events observed by the total number of trials (4 × our sample size for this particular condition). We then used *p* to calculate $P\left(k\right)$, i.e. the probability of getting *k* stings out of four trials (so with *k* being 0, 1, 2, 3 or 4 stings), using the binomial probability formula:


P(k)=(4k)pk(1−p)4−k.

We compared the values obtained from this theoretical calculation with our observations using *χ*^2^ tests.

To further investigate if bees were consistent across trials (figure 2*b,d,f,h*), we calculated the probability of bees stinging in both the first half of the experiment (trial 1 and/or 2, event *A*) and the second half (trial 3 and/or 4, event *B*) by chance, i.e. under the assumption that these events are independent: P(A∩B)=P(A)×P(B) with *P*(*A*) and *P*(*B*) the overall stinging frequencies for these trials observed from our data. The consistency to sting was defined as *P*(*B|A*) and calculated with the formula $P\left(B|A\right)=P(A\cap B)/P(A)$ using either the observed or the theoretical value for P(A∩B). The observed and theoretical consistencies were then compared using a *χ*^2^ test. A similar analysis was performed using non-stinging responses to calculate and evaluate the consistency to not sting.

**Figure 3.**
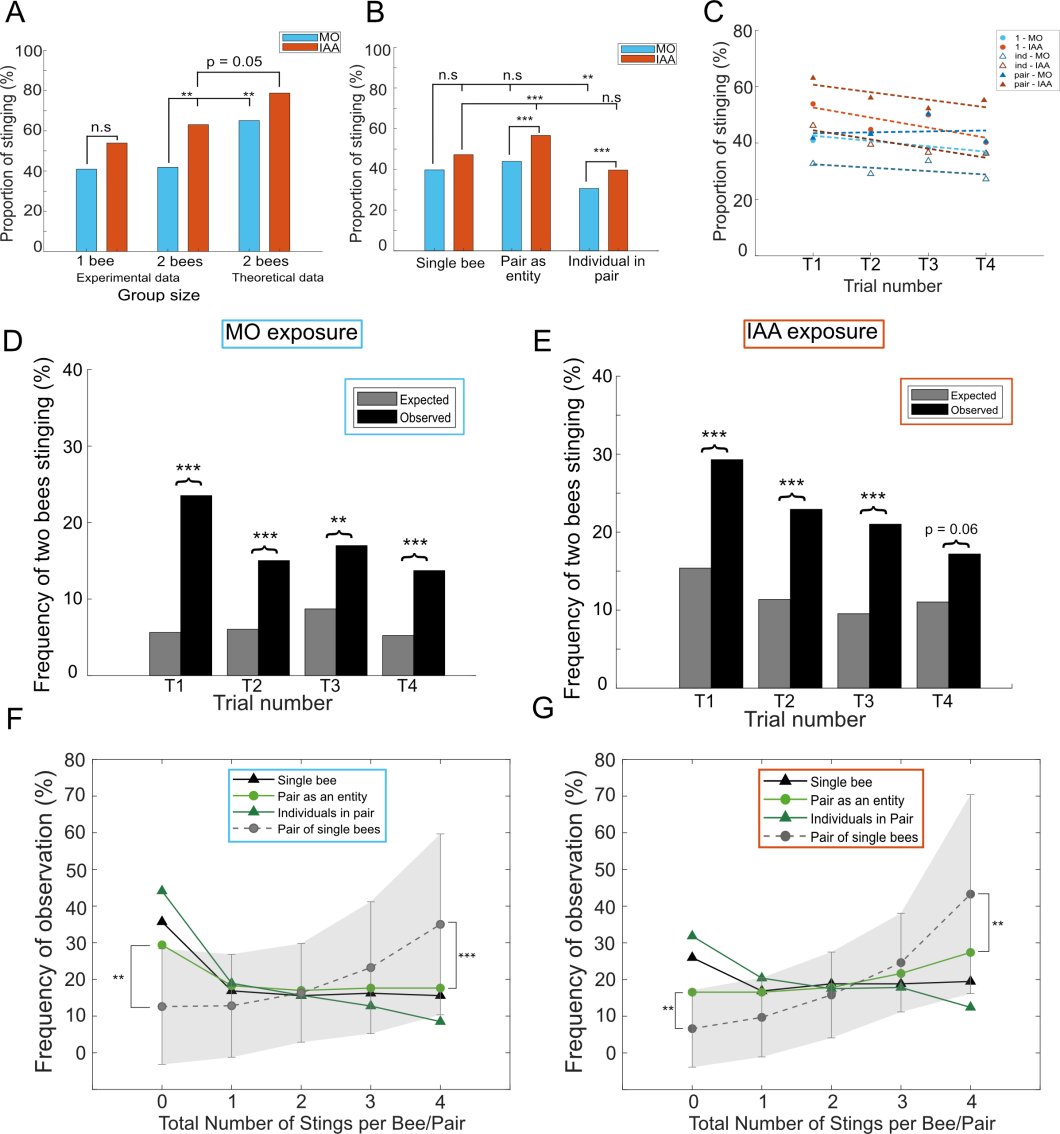
Alarm pheromone responsiveness depends on group size and decreases over trials. (*a*) Stinging probability in the first trial, as a function of odour exposure (MO or IAA) and group size (single bees or pairs of bees). The theoretical data represent the results expected from pairing single bees. Comparisons were performed using *χ*^2^ tests. (*b*) Stinging probability averaged over all trials, as a function of odour exposure (MO or IAA) and social context (single bees, pairs as entities and individuals within pairs). Generalized linear mixed-effect models, interaction between odour and social context. (*c*) Stinging probability decreases over trials in the presence of IAA, but not MO, in all social contexts. Generalized linear mixed-effect models, three-way interaction between odour, social context and trial. (*d*) Observed (black) versus expected (grey) frequencies of both bees in a pair stinging together in the presence of MO. The expected frequency assumes that the bees act independently from each other (no internal recruitment). (*e*) Same as (*d*) but in the presence of IAA. (*f*) Distribution of stings per bee in the presence of MO, in four social contexts: single bees (individual outcome), pairs considered as an entity (collective outcome), individuals in pairs (individual outcome) and pairs of single bees (collective outcome, data generated *in silico*). Pairwise comparisons on the proportions of bees never (0 sting) and always (4 stings) were done using *χ*^2^ tests. (*g*) Same as (*f*) but in the presence of IAA. n.s. *p* > 0.05, *p < 0.05, **p < 0.01, ***p < 0.001.

To better understand how bees interacted within a pair (figure 3), we considered either the ‘pair as an entity’ or ‘individuals within the pair’. If at least one bee in the pair was observed to be stinging, we considered the ‘pair as an entity’ to be stinging, whereas the stinging scores of both individuals were considered independently for ‘individuals within the pair’. In addition, we created artificial data by pairing two single bees (i.e. from 1-MO or 1-IAA) in all possible combinations and looking at their joint outcome.

We used generalized mixed-effect models to comprehensively analyse how stinging responses were shaped by the different social factors and by repeated testing. This part of the analysis was performed in R v4.3.2 [30] with the ‘lme4’ package [31]. We created two datasets: one including the data from single bees and the responses of pairs considered as an entity (with factors odour, trial, group size, colony of origin and bee/pair ID) and one with the single bees and the responses of all individuals within the pairs (with factors odour, trial, alone/in pair, colony of origin and bee ID). For both datasets, colony of origin and entity ID were included as random effects and the other factors as fixed effects. Model comparisons (see electronic supplementary material, statistical information) demonstrated that all factors accounted for significant variation in the data; hence, we used full models with interactions for both datasets. *Post hoc* comparisons were performed using the functions *emmeans* for the interaction between group size or alone/in pair and odour and *emtrends* for the three-way interaction including trial as a continuous variable, with the Sidak method to adjust *p*-values [32].

We also looked for internal recruitment via alarm pheromone release within the pair of bees (figure 3*d*,*e*), by calculating the probability of two bees stinging together independently and comparing that to our observations. To do so, we used the binomial law again and first estimated the stinging probability *p* of a bee in the absence of SAP being released, by looking at the frequency of trials in which none of the bees stung: $P\left(0\right)={\left(1-p\right)}^{2}$, so p=1-√P(0), with *P*(0) the observed proportion of trials in which none of the bees stung. We then used this value *p* to calculate the theoretical $P\left(1\right)=2p\left(1-p\right)$ and $P\left(2\right)=p^{2}$ in which *P*(1) is the proportion of trials with only one out of the two bees stinging and *P*(2) the proportion of trials with both bees stinging. These theoretical values were compared to our observations using *χ*^2^ tests. We confirmed the decrease in stinging likelihood for internal recruitment using Pearson’s tests.

#### 2.3.2. Group composition

**Figure 4.**
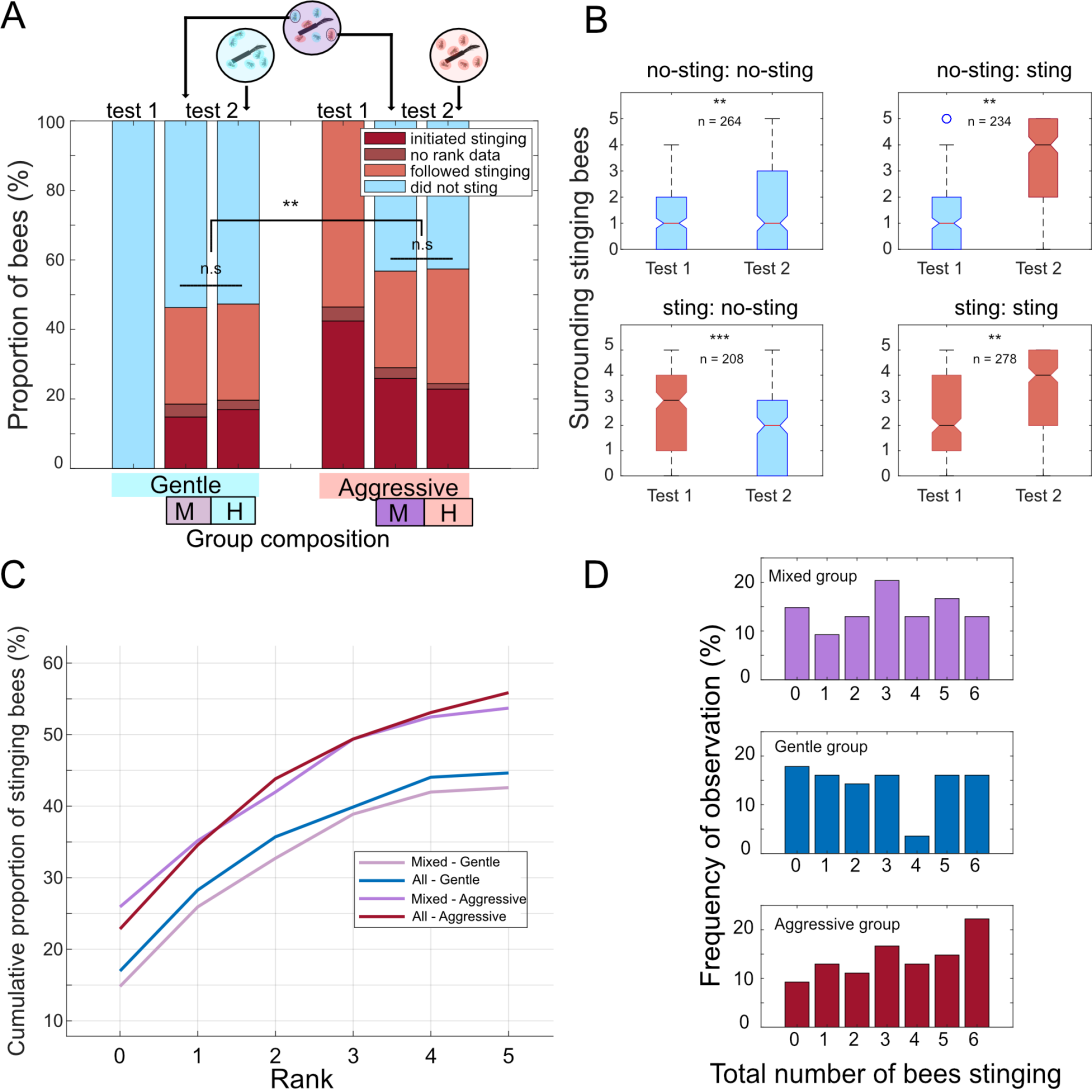
Group composition does not matter but affect the collective outcome. (*a*) Bees were classified as gentle or aggressive based on test 1 (data plotted for reference). Proportion of gentle/aggressive bees stinging (detailed between initiators and followers) or not depending on the composition of the group they were placed for the second test (M: mixed group, H: homogeneous group). Note that the mixed group is split, since we considered separately the behaviour of gentle bees and the behaviour of aggressive bees within this group. Comparisons were performed using *χ*^2^ tests. (*b*) The number of bees stinging (and hence emitting SAP) in the arena just before the focal bee stung, or at the end of the trial if the bee did not sting. The bees are split into four categories depending on their behaviour in both tests (no sting–no sting; no sting–sting; sting–no sting; sting–sting). (*c*,*d*) Distribution of stings per group for the three group types. These distributions are all significantly different from one another. n.s. *p* > 0.05, *p < 0.05, **p < 0.01, ***p < 0.001.

Our group composition dataset consisted of the stinging score (1 or 0), the rank (0–6), stinging response time (0–600 seconds) and the number of surrounding stinging bees around the focal individual bee within a group, in the first and the second tests. To test whether group composition has an effect on focal bees, we calculated the stinging average of each group, i.e. gentle bees in the mixed group, aggressive bees in the mixed group, the all-gentle bees group and the all-aggressive bees group (figure 4*a*). We compared these proportions by using the two-proportion *Z*-test.

We also looked into the effect of surrounding stinging bees on a focal bee, by comparing each group of focal bees in test 1 (T1) and test 2 (T2), regardless of the group composition (figure 4*b*). This resulted in four categories of focal bees: B00, bees that did not sting in T1 and T2; B01, bees that did not sting in T1 but did in T2; B10, bees that stung in T1 but not in T2; and B11, bees that stung in both T1 and T2. For each category, we split the number of surrounding bees (excluding the focal bee) into T1 and T2 and visualized it using box plots. The difference between each of the box plots within each category was compared using Wilcoxon signed rank tests.

To further assess if individual classification and/or group composition could predict the stinging behaviour of bees during test 2, we counted the number of bees from each of these four categories that attacked with rank 0, 1, etc. (corresponding to the number of SAP units in the arena at the time that they made the decision to sting) and plotted this as a cumulative distribution (figure 4*c*). These curves were then compared using generalized linear models with the (cumulated) percentage of stinging bees as the response variable and rank, bee type (aggressive or gentle based on test 1) and group type (mixed, all-gentle, all-aggressive) as explanatory variables (see also electronic supplementary material).

We compared the differences in the collective output of the different group compositions (figure 4*d*) by performing *χ*^2^ tests on each pair of groups (all-aggressive versus mixed; all-aggressive versus all-gentle; and mixed versus all-gentle).

## 3. Results

### 3.1. Individual bees vary in their likelihood to sting

Our first aim was to establish whether individual honeybees show variability in their stinging behaviour. To do so, we started by testing single bees repeatedly in a stinging assay, with no odour (just a solvent control, condition 1-MO). The overall probability of stinging that our population of bees exhibited in this condition was 0.4. We used this value to calculate the theoretical distribution that our data would have if all bees had this same likelihood to sting and compared it to our observations (figure 2*a*; see §2.3.1 for more details on this calculation). Our data deviated significantly from this theoretical distribution (*χ*^2^ = 126.64; *df* = 4, *p* < 0.001; *n* = 154). In particular, there were more bees never stinging (*χ*^2^ = 59.5529; *df* = 1, *p* < 0.001) and more bees always stinging (*χ*^2^ = 105.3257; *df* = 1, *p* < 0.001) than could be expected from a homogeneous population with a likelihood to sting of 0.4. By contrast, fewer bees stung once or twice than expected (1 sting: *χ*^2^ = 14.15; *df* = 1, *p* < 0.001; 2 stings: *χ*^2^ = 15.88; *df* = 1, *p* < 0.001). Thus, there was a significant heterogeneity in our population.

Could we further predict the behaviour of an individual bee based on initial observations? To check this, we counted the number of bees that stung in the first half of the experiment (trial 1 and/or trial 2) and used conditional probability rules to determine how many of them would also sting in the second half of the experiment (trial 3 and/or trial 4) if these two events were independent (figure 2*b*; see §2.3.1 for details). We observed that stinging consistency in this condition was 75%, which was significantly higher than the theoretical value of 50.64% (*χ*^2^ = 11.70; *df* = 1; *p* < 0.01). Similar predictability was observed with respect to the decision to not sting, with 87.9% of bees being consistent (instead of 49.35% expected by chance; *χ*^2^ = 30.21; *df* = 1; *p* < 0.01). Thus, bees were likely to exhibit the same behaviour in the second half of the experiment as they did in the first one. For further experiments (including our group composition experiment presented below), it would be useful to know if a single trial is enough to classify a bee as a stinger or a non-stinger. Hence, we did the same analysis but from trial 1 to trial 2. We observed that the predictability in the decision to sting was 73.01% (significantly higher than the expected 43.5%, *χ*^2^ = 20.01; *df* = 1; *p* < 0.01) and the predictability with the decision to not sting was 76.92% (significantly higher than the expected 56.5%, *χ*^2^ = 7.38; *df* = 1; *p* < 0.01).

### 3.2. Social factors do not affect stinging consistency

Next, we investigated if variability in stinging decisions changed in the presence of social cues, i.e. if social cues could either destabilize or cement the choice made by an individual. We evaluated the effect of two types of social cues: the SAP in the form of its main component IAA (condition 1-IAA) or the presence of a social partner (pair-MO). We also looked at the interaction between these two cues in the pair-IAA condition.

In all cases, we found similar results as for the 1-MO condition. When looking at total number of stings per bee/pair, the data distribution did not fit that of a theoretical, homogeneous population for any of the conditions (1-IAA: figure 2*c*, *χ*^2^ = 98.99; *df* = 4; *p* < 0.001; pair-MO: figure 2*e*, *χ*^2^ = 106.21; *df* = 4; *p* < 0.001; pair-IAA: figure 2*g*, *χ*^2^ = 89.11; *df* = 4; *p* < 0.001). Compared to the expected distribution, there was always an excess of bees never or always stinging, and a deficit of bees with more variable behaviour (detailed statistics in figure 2). Stinging consistency between the first and the second half of the experiment ranged between 73.74 and 80.23%, while the consistency to not sting ranged between 74.16 and 80.73%. All of these values were significantly higher than could be expected under an assumption of independence, except the consistency to sting in the pair-IAA condition (figure 2*d*,*f*,*h*). Note that the data presented here considered the pair as a unit producing a collective output, but that similar results were obtained when considering the individual bees within the pair (electronic supplementary material, figure S1).

Since we used several colonies to collect these data (10 in total; see electronic supplementary material, table S1), it was important to check that the stable inter-individual variability that we describe here was not due to an inter-colony variability. We provide an overview of the response patterns observed per colony in electronic supplementary material, figures S3 and S4; however, our samples sizes did not allow statistical comparisons within each colony. As a way of getting around this issue, we included both bee (or pair) ID and colony of origin as random factors in a generalized mixed-effect model on our full dataset (see §2.3.1 for details). The identity of the bee/pair accounted for five times more of the variance than the colony of origin (see electronic supplementary material, statistical information). This confirmed that the variability observed here was not principally driven by colony differences.

### 3.3. Responsiveness to the alarm pheromone depends on social context

One of the social signals that we considered was the SAP (in the form of its main component, IAA) which is expected to recruit bees into stinging [16,17]. However, it was shown that this effect is only marginal in single bees, possibly due to an already higher aggressiveness during baseline (solvent) conditions [29]. Analysing the first trial alone revealed a similar pattern as published earlier: there was only a trend for IAA to increase stinging likelihood in single bees, whereas this effect was significant for pairs of bees (figure 3*a*; single bees: *χ*^2^ = 3.38, *p* = 0.06; pairs: *χ*^2^ = 9.03, *p* < 0.01). In addition, the real pairs were less aggressive than could be expected from pairing single bees both under baseline conditions (*χ*^2^ = 8.27, *p* < 0.01) and in the presence of IAA (*χ*^2^ = 3.86, *p* = 0.049).

To look deeper into this phenomenon, we used generalized linear models and looked at the interaction (i) between either odour and group size when considering the pair as a unit producing a collective output or (ii) between odour and social context (i.e. alone/in pair) when considering each individual independently (figure 3*b*; note that this time all four trials were considered). *Post hoc* pairwise comparisons confirmed that single bees did not respond to IAA (*z* = −2.018, *p* = 0.23), whereas pairs as entities did (*z* = −4.327, *p* < 0.001). Similar results were obtained when considering the IAA responsiveness of the individual bees within pairs (*z* = −3.68, *p* < 0.001).

### 3.4. Responsiveness to the alarm pheromone decreases over repeated trials

We also checked if the stinging likelihood of bees was affected by repeated testing, by looking at the three-way interaction between trial, odour and social context (since we already know that both of these latter factors influence stinging likelihood). In all three of the social contexts considered (single bee, pair as an entity and individual within pair), the proportion of bees stinging within each trial was stable across the four trials under baseline conditions (figure 3*c*; 1-MO: *z* = −1.03, *p* = 0.29; pair-MO: *z* = 0.67, *p* = 0.86; ind-MO: *z* = −1.35, *p* = 0.17). This was an important result, as it showed that honeybees did not change their evaluation of our test stimulus (the rotating dummy) even after repeated exposure. In contrast, the stinging frequency of bees exposed to IAA decreased across trials (figure 3*c*; 1-IAA: *z* = −2.59, *p* < 0.05; pair-IAA: *z* = −1.46, *p* = 0.14; ind-IAA: *z* = −3.45, *p* < 0.001). This suggests that IAA lost its value as an alerting signal over repeated exposures.

When a bee stings the dummy, it also releases SAP by extruding its stinger. When bees are tested in pairs, this may recruit the second bee. To test this, we first calculated the probability to sting of the first bee, which can be obtained by looking at the proportion of trials in which none of the bee stung (see §2.3.1 for details). From this value, we then calculated the theoretical probability of the bees to sting together assuming independence (i.e. no recruitment; grey bars in figure 3*d,e*). In nearly all trials and for both MO and IAA conditions, the proportion of bees stinging together was higher than expected by chance, confirming that internal recruitment did happen (MO: figure 3*d*, trial 1: *χ*^2^ = 56.88, *p* < 0.001, trial 2: *χ*^2^ = 13.35, *p* < 0.001, trial 3: *χ*^2^ = 7.86, *p* < 0.01, trial 4: *χ*^2^ = 12.59, *p* < 0.001; IAA: figure 3*e*, trial 1: *χ*^2^ = 12.59, *p* < 0.001, trial 2: *χ*^2^ = 11.78, *p* < 0.001, trial 3: *χ*^2^ = 13.82, *p* < 0.001, trial 4: *χ*^2^ = 3.43, *p* = 0.06). Notably however, recruitment decreased over trials and this was significant in the presence of IAA (MO: *r* = −0.81, *p* = 0.18; IAA: *r* = −0.97, *p* < 0.05). Thus, our bees also stopped responding to the complete SAP blend naturally released by a nestmate, not only to synthetic IAA as demonstrated previously (figure 3*c*).

### 3.5. The presence of another bee reduces the likelihood to sting

We already hinted at the fact that bees did not have the same stinging probability when tested alone or in pairs by pointing out that pairs of bees were less aggressive than could be expected from pairing single bees (see text above and figure 3*a* on the first trial). This was also confirmed by the *post hoc* pairwise comparisons of our generalized linear models: in the control (MO) condition, pairs (as entities) had a similar likelihood to sting as single bees (*z* = −1.44, *p* = 0.62; figure 3*b*), which meant that individuals within each pair were less likely to sting than single bees (*z* = −3.04, *p* < 0.01). As a consequence of both this group size effect and the change in responsiveness to IAA described above, the stinging likelihood in the presence of IAA was similar for individual in pairs than for single bees (*z* = 2.38, *p* = 0.99), but increased for pairs considered as an entity (*z* = −3.22, *p* < 0.001).

When looking at the full distribution of our data rather than at the average over all trials, the behaviour of single bees was similar to the collective output of pairs in either odour condition (MO: figure 3*f*, *χ*^2^ = 0.68; *df* = 4, *p* = 0.95; IAA: figure 3*g* *χ*^2^ = 1.61; *df* = 4, *p* = 0.80). It was also not distinguishable from the behaviour of individuals within the pairs (MO: figure 3*f*, *χ*^2^ = 3.22; *df* = 4, *p* = 0.52; IAA: figure 3*g*, *χ*^2^ = 1.08; *df* = 4, *p* = 0.89). In both odour conditions, however, the distribution tended to be slightly shifted towards lower number of stings for the individuals within the pairs, whereas the pairs collectively tended to attack slightly more often than single bees. This pattern became more apparent when we computed the collective output that could be expected from pairing single bees (see §2.3.1 for details). Compared to the real pairs, this theoretical distribution tended to be shifted towards higher stinging frequencies, as confirmed by an excess of bees stinging four times and a deficit of bees never stinging (MO: figure 3*f*, overall: *χ*^2^ = 6.98, *df* = 4, *p* = 0.13, 0 stings: *χ*^2^ = 9.60; *df* = 1, *p* < 0.01, 4 stings: *χ*^2^ = 17.10; *df* = 1, *p* < 0.001; IAA: figure 3*g*, overall: *χ*^2^ = 4.60; *df* = 4, *p* = 0.33, 0 stings: *χ*^2^ = 5.96; *df* = 1, *p* < 0.05, 4 stings: *χ*^2^ = 9.22; *df* = 1, *p* < 0.01).

Overall, our results were in agreement with a previous study showing that group size acts as a negative feedback on honeybee stinging behaviour [19]. The remarkable similarity between the output of pairs and single bees was reminiscent of a homeostatic process.

### 3.6. Stinging likelihood is also influenced by an internal state

Individual bees varied in their likelihood to sting, with some bees stinging in all trials and some never stinging. A large number of bees, however, exhibited more nuanced behaviours by stinging 1, 2 or 3 times out of the four trials. We wondered if some finer structure could be found that would partly explain these stinging patterns. In particular, we postulated that the likelihood to sting might be modulated by an internal state of the bee. If this was true, then trials closer in time should be more correlated than trials further apart (as a bee would be less likely to change state within a shorter duration). We counted the number of times that bees showed the same behaviour (i.e. stinging | stinging or non-stinging | non-stinging) versus switched behaviours (i.e. stinging | non-stinging or non-stinging | stinging) between either consecutive or distant trials (electronic supplementary material, figure S2; table 1). As expected, bees were less likely to switch between consecutive trials than between trials separated by longer durations (*χ*^2^ = 9; *df* = 1; *p* < 0.01).

**Table 1.** Total number of bees performing the same behaviour or switching behaviour in consecutive or distant trials (see also elctronic supplementary material, figure S2). *χ*^2^ = 15; *df* = 1; **p < 0.01. Thus, the bees changed their decision more often between distant than consecutive trials.

	consecutive trials	distant trials
same	480	397
switch	504	587
% of switches	51.28	59.59

Finally, we classified the observed stinging patterns according to the underlying stinging likelihood (i.e. 0, 0.25, 0.5, 0.75 or 1; electronic supplementary material, table S3). Within each of these categories, all sequences should be equally likely if there is no temporal component to stinging behaviour. This was true for most sequences, but a few exceptions stood out (highlighted in dark yellow). First, the sequences 1−0−0−0 and 1−1−0−0 were over-represented, but only for bees tested with IAA. This was not surprising given that we already found that responsiveness to IAA decreases over trials. On the other hand, the sequences 0−1−0−1 and 1−0−1−0 tended to be under-represented. These sequences were characterized by the highest instability, reflecting bees that switch their behaviour in between every single trial. The fact that they were not likely was consistent with a state-dependence.

### 3.7. Group composition does not matter for focal bees

After establishing that bees were likely to maintain their stinging decision from one trial to the next provided that the context was similar, we performed our ‘group composition’ experiment (see §2.2; figure 1*c,d,e*). Our aim here was to determine whether individual bees were influenced by the phenotype of their nestmates (low or high stinging likelihood) when taking part in an attack. Our approach was to first classify individuals as aggressive or gentle based on their response during a first trial, then create groups of known composition (all-gentle, all-aggressive or half–half) and test the bees again in this new social context. We hypothesized that we might observe behavioural flexibility among individuals in the group such that the number of stinging bees (collective output) would remain stable. This would translate to bees changing their decision to sting more readily in the homogeneous groups than in the mixed one, to achieve some middle ground. However, this was not what we observed. Bees that were defined as ‘gentle’ during test 1 were equally likely to sting during test 2, whether they were in the mixed group or in the all-gentle group, and similarly for ‘aggressive’ bees in the mixed or all-aggressive groups (figure 4*a*). This leads us to conclude that group composition did not affect the stinging decision of individual bees.

A striking feature of the second trial was that many bees switched in their decision to sting or not compared to the first trial (figure 4*a*). To better understand this variability, we looked at the SAP concentrations (defined by the number of surrounding stinging bees, so that each stinging bee releases one unit of SAP) inside the arena during each trial, for bees that either switched in their stinging behaviour or not (figure 4*b*). We found that on average, bees that stopped stinging in the second trial were also exposed to less SAP (*z* = 5.23; *p* < 0.001), whereas bees that started stinging were exposed to more SAP than in the first trial (*z* = −11.35; *p* < 0.01). Bees that remained gentle were exposed to slightly higher SAP levels in the second test (*z* = −2.61; *p* < 0.01), while bees that remained aggressive were actually exposed to even more SAP during the second trial (*z* = −6.99; *p* < 0.01). Thus recruitment (or lack thereof) via SAP being released likely explained a large part of the behavioural variability observed.

Despite this component of social modulation, stable inter-individual differences were still apparent in this experiment. Consistent with our classification, aggressive bees were overall more likely to sting than gentle ones (figure 4*a*; *z* = 3.2063, *p* < 0.01), and stung faster (electronic supplementary material, figure S6). This difference was obvious also when breaking down the attack into its successive waves of recruitment by ranking the bees (figure 4*c*; electronic supplementary material, figure S6). Comparisons of generalized linear models fitted to these data (figure 4*c*) confirmed that the classification of the bees as gentle or aggressive based on test 1 was a predictor of their behaviour during test 2 (ANOVA, *F* = 46.469, *p* < 0.001) but not the type of group they were placed in (ANOVA, *F* = 0.378, *p* = 0.821; see the electronic supplementary material for more detailed statistical information). As a result, the collective outputs of each group type were significantly different (figure 4*d*; all-aggressive group versus mixed group: *χ*^2^ = 11.33; *df* = 1; *p* < 0.05; all-aggressive versus all-gentle group: *χ*^2^ = 32.61; *df* = 1; *p* < 0.05; all-gentle group versus mixed group: *χ*^2^ = 29.99; *df* = 1; *p* < 0.05). Among mixed groups, the data distribution was centred on three bees stinging, which is interesting since that is the number of aggressive bees that we put in. By comparison, the all-gentle groups were most likely to not attack (0 bee stinging), while the data distribution of all-aggressive groups was strongly biased towards high numbers of stinging bees.

## 4. Discussion

In ‘The bear and the bees’, a fifteenth-century cautionary tale about the danger of giving way to anger, a bear overturns beehives after being stung by a bee and is consequently chased by thousands of them. This is but one example of the prominence of honeybee mass-stinging behaviour. This collective defensive response has been studied mainly at the colony level, yet the decision to sting (or not) has to be taken by each bee individually. In this study, we brought the focus back to individual bees, within the context of the collective. We explored the within-individual consistency and between-individual variability of stinging responses, and how they might be affected by social factors.

We first showed that individual bees vary in their likelihood to sting. It is worth noting that this variability was evident even though we specifically tested ‘defensive bees’ that attacked a black feather waved in front of the hive entrance. This was likely a rather homogeneous population consisting of middle-aged guard bees and maybe older foragers or soldiers [3,4]. Sampling the full spectrum of bees composing the colony (by also catching younger nurse bees for example) would likely make this inter-individual variability even stronger. Importantly, individual differences accounted for most of the variation in our data, not colony of origin. Individuality in stinging behaviour thus persists despite the strong influence of social context that was demonstrated by earlier studies [12–14]. Taken together, these results suggest that the neurophysiological bases for stinging propensity may be accessible by comparing ‘gentle’ and ‘aggressive’ bees of the same colony. It will be especially powerful to compare individuals at the same life stage, since they should exhibit otherwise similar profiles.

We also studied the impact of social factors, namely the presence of the main alarm pheromone component IAA, of another bee or both, on the stinging responses of individuals. Predictably, the presence of IAA increased the likelihood to sting but it is interesting that it did so without affecting individual consistency. This supports the hypothesis that IAA acts primarily by lowering the stinging threshold of bees [29]. On the other hand, the presence of another bee reduced the likelihood to sting. This is a strikingly robust result, as it is now the third time that we have replicated it [19,29]. This effect may seem counter-intuitive, given the general belief that larger honeybee colonies are more aggressive. However, there is very little scientific evidence to support this claim. On the contrary, Collins & Kubasek [33] reported that ‘the number of stings in the target was negatively correlated with population and [hive] volume’. In addition, most field tests do not evaluate aggressiveness per bee. An overall increase in the number of stinging bees may not be proportional to the increase in population, resulting in a decrease in aggressiveness *per capita*. Such a phenomenon has been demonstrated for wasps and stingless bees [34,35]. Individual consistency was again not affected, confirming that this group size effect takes the form of negative feedback that raises the stinging threshold. These opposing forms of social feedback (positive via the alarm pheromone, negative via group size) interact to regulate the stinging decisions of individual bees, reflecting the fine balance between the benefits and costs of defence. Finally, we also replicated the finding that IAA responsiveness is higher in pairs of bees than in individuals [29]. It may be that responses to this social signal are gated by the presence of social partners which could release this pheromone. Since IAA is also produced by some plants [36], such a mechanism could ensure that the stinging response is tied to the relevant context. In ants, aggressiveness towards non-nestmates decreases after isolation but remains high just after the separation [37]. It would be interesting to check if this is also the case for honeybees.

In addition to confirming previous results, this study also revealed that the IAA-induced stinging responsiveness of honeybees decreases over trials. Alaux *et al.* [38] obtained similar results by measuring either the number of bees recruited at the colony entrance or the number of fanning bees in cages over repeated exposures to IAA (and with similar timescales). This loss of responsiveness was detectable across trials both with synthetic IAA and with the full SAP released by some of the bees tested (internal recruitment). This happened despite relatively short durations of exposure (3 minutes) followed by long recoveries (30–40 minutes). This makes it unlikely that peripheral sensory adaptation was involved, although this would have to be formally demonstrated. Indeed, bees start recovering from much longer term exposure to IAA within 60 minutes [39]. By contrast, responsiveness to the dummy itself did not seem to be affected by repeated exposures. These results are somewhat surprising and suggest that these two types of stimuli are processed by different circuits in the bee brain. In the context of foraging, bees are known to use social information only when their own information is outdated [40], uncertain [41] or when errors are costly [42]. That bees stop responding to the SAP but not to the threat itself might be another example of social information being less valued than personal information. On top of these biological considerations, establishing to which extent repeated testing of individual stinging responses is possible is an important outcome of this study, as it opens the door to many possible experiments. For example, one could test the same bees before and after a treatment, or with and without a putative modulatory factor, rather than relying on between-groups comparisons.

While many bees were fully consistent in their decision to sting or not, some were more variable. We interpret these bees as having an evaluation of the threat presented by the dummy close to their stinging threshold, such that their decision could easily switch depending on other factors. At least one of these factors appears to be internal, as demonstrated by the temporal correlation across trials. But what this internal state might be and by which mechanism it modulates stinging responses remain to be discovered. Our bees were fed ad libitum in between trials, so hunger is not likely. In recent years, it has become apparent that brain function is an active process rather than an input/output transformation, that sometimes creates noise [43]. We can but wonder if the internal state modulation that we observe here is a by-product of other ongoing physiological functions, or an actively produced variability. Such a mechanism might be useful to achieve division of labour during colony defence, for example.

Finally, we found no effect of group composition on the individual likelihood to sting. In an early study, Lecomte reported that after removing the most aggressive bees from a group of caged bees, the other bees became more aggressive towards the dummy [20]. His tentative conclusion was that aggressive bees inhibited the responses of the other bees. If this was true, we would have expected our gentle bees to sting less often in the mixed groups than in the all-gentle groups, but this was not the case. We propose that his results were rather the consequence of the reduced group size, which was a confounding factor in his experimental design. An effect of group composition was also found in a study mixing bees from disturbed and undisturbed colonies, which had different aggression levels as a result of the disturbance [21]. It concluded that ‘disturbed bees increase their aggression levels to compensate for a shortage of high-aggression individuals’ when no undisturbed bees were included. This study was different from ours in that it used the intruder assay, in which a non-nestmate is introduced into a group of caged bees, rather than a stinging assay. It may be that social inhibition occurs for the task of deterring non-nestmates because it does not require a collective response (one bee is enough to do the job).

Our results suggest that bees do not modulate their stinging behaviour based on the decisions of their current social partners, except via SAP recruitment. As mentioned earlier, however, we sampled only defensive bees which were likely at a similar stage in their behavioural maturation. It might be interesting to repeat this experiment with bees from more diverse populations, e.g. nurses and foragers. These bees are known to carry different cuticular hydrocarbon profiles [44,45] which could provide a basis for their identification, in addition to varying in their stinging likelihood [14,27].

Overall, our study demonstrates that stable inter-individual variability in stinging behaviour exists and should be amenable to further study of its (neuro)physiological correlates. It supports the idea that social factors such as the SAP or the presence of nestmates act by shifting the stinging threshold of individuals and uncovers the existence of an internal modulation of this threshold. Intriguingly, bees seem to consider the SAP as an altogether unreliable signal, as shown by their loss of responsiveness over trials and by gating of this response by the presence of at least one other bee. The mechanisms behind all these effects remain to be addressed in future studies.

## Data Availability

The data have been deposited on Dryad [46]. Supplementary material is available online [47].

## References

[1] Dall SRX, Bell AM, Bolnick DI, Ratnieks FLW. 2012 An evolutionary ecology of individual differences. Ecol. Lett. **15**, 1189–1198. (10.1111/j.1461-0248.2012.01846.x) 22897772 PMC3962499

[2] Robinson GE. 1992 Regulation of division of labor in insect societies. Annu. Rev. Entomol. **37**, 637–665. (10.1146/annurev.en.37.010192.003225) 1539941

[3] Moore AJ, Breed MD, Moor MJ. 1987 The guard honey bee: ontogeny and behavioural variability of workers performing a specialized task. Anim. Behav. **35**, 1159–1167. (10.1016/s0003-3472(87)80172-0)

[4] Breed MD, Robinson GE, Page RE. 1990 Division of labor during honey bee colony defense. Behav. Ecol. Sociobiol. **27**, 395–401. (10.1007/bf00164065)

[5] Hunt GJ. 2007 Flight and fight: a comparative view of the neurophysiology and genetics of honey bee defensive behavior. J. Insect Physiol. **53**, 399–410. (10.1016/j.jinsphys.2007.01.010) 17379239 PMC2606975

[6] Guzmán-Novoa E, Page RE. 1999 Selective breeding of honey bees (Hymenoptera: Apidae) in Africanized areas. J. Econ. Entomol. **92**, 521–525. (10.1093/jee/92.3.521)

[7] Arechavaleta-Velasco ME, Hunt GJ. 2004 Binary trait loci that influence honey bee (Hymenoptera: Apidae) guarding behavior. Ann. Entomol. Soc. Am. **97**, 177–183. (10.1603/0013-8746(2004)097[0177:btltih]2.0.co;2)

[8] Arechavaleta-Velasco ME, Hunt GJ, Emore C. 2003 Quantitative trait loci that influence the expression of guarding and stinging behaviors of individual honey bees. Behav. Genet. **33**, 357–364. (10.1023/a:1023458827643) 12837024

[9] Hunt GJ, Guzmán-Novoa E, Fondrk MK, Page RE. 1998 Quantitative trait loci for honey bee stinging behavior and body size. Genetics **148**, 1203–1213. (10.1093/genetics/148.3.1203) 9539435 PMC1460054

[10] Guzmán-Novoa E, Hunt GJ, Uribe JL, Smith C, Arechavaleta-Velasco ME. 2002 Confirmation of QTL effects and evidence of genetic dominance of honeybee defensive behavior: results of colony and individual behavioral assays. Behav. Genet. **32**, 95–102. (10.1023/a:1015245605670) 12036115

[11] Shorter JR, Arechavaleta-Velasco M, Robles-Rios C, Hunt GJ. 2012 A genetic analysis of the stinging and guarding behaviors of the honey bee. Behav. Genet. **42**, 663–674. (10.1007/s10519-012-9530-5) 22327626

[12] Avalos A *et al*. 2020 Genomic regions influencing aggressive behavior in honey bees are defined by colony allele frequencies. Proc. Natl Acad. Sci. USA **117**, 17135–17141. (10.1073/pnas.1922927117) 32631983 PMC7382227

[13] Guzmán-Novoa E, Page RE Jr. 1994 Genetic dominance and worker interactions affect honeybee colony defense. Behav. Ecol. **5**, 91–97. (10.1093/beheco/5.1.91)

[14] Paxton RJ, Sakamoto CH, Rugiga FCN. 1994 Modification of honey bee (Apis mellifera L.) stinging behaviour by within-colony environment and age. J. Apic. Res. **33**, 75–82. (10.1080/00218839.1994.11100853)

[15] Traniello IM *et al*. 2023 Single-cell dissection of aggression in honeybee colonies. Nat. Ecol. Evol. **7**, 1232–1244. (10.1038/s41559-023-02090-0) 37264201

[16] Boch R, Shearer DA, Stone BC. 1962 Identification of iso-amyl acetate as an active component in the sting pheromone of the honey bee. Nature **195**, 1018–1020. (10.1038/1951018b0) 13870346

[17] Nouvian M, Reinhard J, Giurfa M. 2016 The defensive response of the honeybee Apis mellifera. J. Exp. Biol. **219**, 3505–3517. (10.1242/jeb.143016) 27852760

[18] López-Incera A, Nouvian M, Ried K, Müller T, Briegel HJ. 2021 Honeybee communication during collective defence is shaped by predation. BMC Biol. **19**, 106. (10.1186/s12915-021-01028-x) 34030690 PMC8147350

[19] Petrov T, Hajnal M, Klein J, Šafránek D, Nouvian M. 2022 Extracting individual characteristics from population data reveals a negative social effect during honeybee defence. PLoS Comput. Biol. **18**, e1010305. (10.1371/journal.pcbi.1010305) 36107824 PMC9477262

[20] Lecomte J. 1951 Recherches sur le comportement agressif des ouvrieres d’apis mellifica. Behaviour **4**, 60–66. (10.1163/156853951x00034) 12979243

[21] Rittschof CC. 2017 Sequential social experiences interact to modulate aggression but not brain gene expression in the honey bee (Apis mellifera). Front. Zool. **14**, 16. (10.1186/s12983-017-0199-8) 28270855 PMC5335736

[22] Ishikawa Y, Miura T. 2012 Hidden aggression in termite workers: plastic defensive behaviour dependent upon social context. Anim. Behav. **83**, 737–745. (10.1016/j.anbehav.2011.12.022)

[23] Laskowski KL, Chang CC, Sheehy K, Aguiñaga J. 2022 Consistent individual behavioral variation: what do we know and where are we going? Annu. Rev. Ecol. Evol. Syst. **53**, 161–182. (10.1146/annurev-ecolsys-102220-011451)

[24] George EA, Brockmann A. 2019 Social modulation of individual differences in dance communication in honey bees. Behav. Ecol. Sociobiol. **73**. (10.1007/s00265-019-2649-0)

[25] Jolles JW, Boogert NJ, Sridhar VH, Couzin ID, Manica A. 2017 Consistent individual differences drive collective behavior and group functioning of schooling fish. Curr. Biol. **27**, 2862–2868.(10.1016/j.cub.2017.08.004) 28889975 PMC5628957

[26] Wray MK, Mattila HR, Seeley TD. 2011 Collective personalities in honeybee colonies are linked to colony fitness. Anim. Behav. **81**, 559–568. (10.1016/j.anbehav.2010.11.027)

[27] Kolmes SA, Fergusson-Kolmes LA. 1989 Measurements of stinging behaviour in individual worker honeybees (Apis mellifera L.). J. Apic. Res. **28**, 71–78. (10.1080/00218839.1989.11100824)

[28] Robinson GE. 1987 Modulation of alarm pheromone perception in the honey bee: evidence for division of labor based on hormonall regulated response thresholds. J. Comp. Physiol. **160**, 613–619. (10.1007/bf00611934)

[29] Nouvian M, Hotier L, Claudianos C, Giurfa M, Reinhard J. 2015 Appetitive floral odours prevent aggression in honeybees. Nat. Commun. **6**, 10247. (10.1038/ncomms10247) 26694599 PMC4703898

[30] R Development Core Team. 2023 R: a language and environment for statistical computing. Vienna, Austria: R Foundation for Statistical Computing.

[31] Bates D, Mächler M, Bolker B, Walker S. 2015 Fitting linear mixed-effects models using lme4. J. Stat. Softw **67**, 1–48. (10.18637/jss.v067.i01)

[32] Lenth RV. 2017 emmeans: estimated marginal means, aka least-squares means. (10.32614/cran.package.emmeans)

[33] Collins AM, Kubasek KJ. 1982 Field test of honey bee (Hymenoptera: Apidae) colony defensive behavior. Ann. Entomol. Soc. Am. **75**, 383–387. (10.1093/aesa/75.4.383)

[34] London KB, Jeanne RL. 2003 Effects of colony size and stage of development on defense response by the swarm-founding wasp Polybia occidentalis. Behav. Ecol. Sociobiol. **54**, 539–546. (10.1007/s00265-003-0662-8)

[35] Nieh JC, Kruizinga K, Barreto LS, Contrera FAL, Imperatriz-Fonseca VL. 2005 Effect of group size on the aggression strategy of an extirpating stingless bee, Trigona spinipes. Insectes Sociaux **52**, 147–154. (10.1007/s00040-004-0785-6)

[36] PubChem. 2024 Isoamyl acetate: compound summary for CID 31276. See https://pubchem.ncbi.nlm.nih.gov/compound/Isoamyl-acetate.

[37] Kleineidam CJ, Heeb EL, Neupert S. 2017 Social interactions promote adaptive resource defense in ants. PLoS ONE **12**, e0183872. (10.1371/journal.pone.0183872) 28910322 PMC5598949

[38] Alaux C, Robinson GE. 2007 Alarm pheromone induces immediate–early gene expression and slow behavioral response in honey bees. J. Chem. Ecol. **33**, 1346–1350. (10.1007/s10886-007-9301-6) 17505874

[39] Al‐Sa’aD BN, Free JB, Howse PE. 1985 Adaptation of worker honeybees (Apis mellifera) to their alarm pheromones. Physiol. Entomol. **10**, 1–14. (10.1111/j.1365-3032.1985.tb00013.x)

[40] Grüter C, Segers FHID, Ratnieks FLW. 2013 Social learning strategies in honeybee foragers: do the costs of using private information affect the use of social information? Anim. Behav. **85**, 1443–1449. (10.1016/j.anbehav.2013.03.041)

[41] Smolla M, Alem S, Chittka L, Shultz S. 2016 Copy-when-uncertain: bumblebees rely on social information when rewards are highly variable. Biol. Lett. **12**, 20160188. (10.1098/rsbl.2016.0188) 27303053 PMC4938046

[42] Wray MK, Klein BA, Seeley TD. 2012 Honey bees use social information in waggle dances more fully when foraging errors are more costly. Behav. Ecol. **23**, 125–131. (10.1093/beheco/arr165)

[43] Brembs B. 2015 Watching a paradigm shift in neuroscience. Winnower (10.15200/winn.142737.71063)

[44] Kather R, Drijfhout FP, Martin SJ. 2011 Task group differences in cuticular lipids in the honey bee Apis mellifera. J. Chem. Ecol. **37**, 205–212. (10.1007/s10886-011-9909-4) 21271278

[45] Vernier CL, Krupp JJ, Marcus K, Hefetz A, Levine JD, Ben-Shahar Y. 2019 The cuticular hydrocarbon profiles of honey bee workers develop via a socially-modulated innate process. eLife **8**, e41855. (10.7554/elife.41855) 30720428 PMC6382352

[46] Kannan K, Galizia GC, Nouvian M. 2024 Consistency and individuality of honeybee stinging behaviour across time and social contexts. Dryad Digital Repository. (10.5061/dryad.m37pvmdc6) PMC11774586 39881791

[47] Kannan K, Galizia CG, Nouvian M. 2025 Supplementary material from: Consistency and individuality of honeybee stinging behaviour across time and social contexts. Figshare. (10.6084/m9.figshare.c.7611313) PMC11774586 39881791

